# Social approach-avoidance conflict: Behavioural, emotional, and neural correlates along a depression–social anxiety spectrum

**DOI:** 10.1162/IMAG.a.1363

**Published:** 2026-09-10

**Authors:** Francisco Cervantes Constantino, Alejo Acuña, Catalina Fernandez, Michelle Guillen, Laura Mendaro, Antonella Brandani, Sebastián Morales, Alfonso Pérez, Javier Mattos, Enrique Cuña, Margarita García-Fontes, Álvaro Cabana, Jorge L. Armony, Michael Gaebler, Victoria B. Gradin

**Affiliations:** Center for Basic Research in Psychology (CIBPsi), Facultad de Psicología, Universidad de la República, Montevideo, Uruguay; Sección Neurociencias, Facultad de Ciencias, Universidad de la República, Montevideo, Uruguay; Instituto de Fundamentos y Métodos en Psicología, Facultad de Psicología, Universidad de la República, Montevideo, Uruguay; Centro Uruguayo de Imagenología Molecular (CUDIM), Montevideo, Uruguay; Centro Interdisciplinario de Ciencia de Datos y Aprendizaje Automático (CICADA), Universidad de la República, Montevideo, Uruguay; Department of Psychology, McGill University, Montreal, Canada; Department of Neurology, Max Planck Institute for Human Cognitive and Brain Sciences, Leipzig, Germany

**Keywords:** approach-avoidance conflict, social interactions, depression, social anxiety, event-related fMRI

## Abstract

Approach-avoidance conflict, in which the same option entails both rewarding and aversive consequences, has been proposed as a key process in internalizing disorders such as depression and social anxiety. Yet, human paradigms that model approach-avoidance conflict and link it to brain function remain scarce. In particular, it has been emphasized the need for studying this conflict in social contexts. We developed a Social Approach-Avoidance Conflict (SAAC) task that involved a social evaluative demand manipulation, and combined it with functional magnetic resonance imaging (fMRI) in 135 young adults. On each trial, participants decided whether to accept a variable reward at the cost of increasing the presence of more or less demanding judges in a future public speaking evaluation, or to avoid and receive a minimal default reward. Depression and social anxiety (D-SA) symptoms were summarized into a single continuous dimension derived from principal component analysis of psychological questionnaires. Behaviourally, higher payments increased approach, whereas higher judge ranks increased negative affect and avoidance. Higher D-SA scores were associated with reduced positive and heightened negative emotional responses to higher-rank judges, stronger preferences for low-rank judges, and greater avoidance of high-rank judges. Neurally, increasing judge rank recruited the dmPFC/dACC during approach decisions, and higher D-SA symptoms were associated with stronger responses in these regions. Increasing payment during approach engaged dmPFC/vmPFC, PCC, and posterior insula, and these reward-related responses were attenuated at higher D-SA levels. In conclusion, internalizing symptoms of depression and social anxiety are associated with a bias in emotional and behavioural responses when a reward competes with a social evaluative threat. At the neural level, internalizing symptoms were associated with an enhanced aversive/conflict signalling in the dmPFC/dACC, and with reduced reward-related activity. Findings shed light on the mechanisms underlying costly social avoidance in internalizing disorders.

## 1 Introduction

People, like other animals, are fundamentally motivated to seek out beneficial outcomes such as food, safety, mating opportunities, or social reward, while also protecting themselves from potential harm by withdrawing from or defending against stimuli perceived as aversive or threatening (Aupperle et al., 2011; Trew, 2011). Approach-avoidance theories capture this basic organization of motivated behaviour and have long provided a foundational framework for understanding these processes in animals including humans (Corr, 2013; Gray, 1970, 1990).

Crucially, in daily life we often face situations where the same choice can lead both to a reward and to an aversive event. For example, delivering a presentation at work may offer potential social or professional rewards while also provoking anxiety or discomfort. Such situations constitute an *approach-avoidance conflict*, in which individuals must decide whether pursuing a reward is worth the potential cost of an aversive experience (Aupperle et al., 2011; Kirlic et al., 2017).

Approach-avoidance theories, and especially the notion of approach-avoidance conflict, have been proposed as fundamental for the understanding of internalizing disorders such as depression and anxiety (Aupperle & Paulus, 2010; Ironside et al., 2020; Smith et al., 2021). In these disorders, alterations in the approach-avoidance processing systems could lead to suboptimal decision-making processes, characterized by diminished approach and excessive avoidance (Ironside et al., 2020; Smith et al., 2021; Trew, 2011). Reduced approach towards rewarding stimuli aligns closely with anhedonia, a core feature of depression (Whitton & Pizzagalli, 2022) and also relevant in anxiety disorders (Taylor et al., 2022). In turn, excessive avoidance is a transdiagnostic process (Hayes & Hofmann, 2021; Kashdan et al., 2006), central to anxiety disorders (Penninx et al., 2021) and also critical in depression (Aldao et al., 2010; Ferster, 1974; Ottenbreit et al., 2014; Trew, 2011). Importantly, avoidance becomes clinically problematic when it entails the loss of potential rewards (Aupperle et al., 2011). Thus, approach-avoidance conflict paradigms, where reward pursuit competes with avoidance of an aversive stimulus, are especially relevant for understanding pathological avoidance.

Although approach-avoidance conflict has been extensively investigated in animal models using reward–punishment contingencies (Kirlic et al., 2017), quantitative behavioural paradigms for studying this conflict in humans remain relatively limited. Many existing human studies rely on self-report or on experimental designs where approach and avoidance drives do not occur concurrently (Kirlic et al., 2017). In the last decade or so, there has been a growing interest in developing human paradigms that evoke genuine approach-avoidance conflict, and in integrating these tasks with fMRI to examine the neural systems involved (Aupperle et al., 2015; Ironside et al., 2020; Schlund et al., 2011; Schultz et al., 2019).

Neuroimaging research suggests that approach-avoidance conflict engages multiple interacting neural systems (Aupperle et al., 2015; Kirlic et al., 2017). Reward-related aspects of the conflict recruit cortico-striatal circuits, whereas aversive or threatening elements activate regions involved in emotion and threat processing, including the amygdala, insula, cingulate cortex, and prefrontal regions. In addition, conflict monitoring and decision-making processes recruit the dorsal anterior cingulate and dorsomedial prefrontal cortex (dACC/dmPFC), and the dorsolateral prefrontal cortex. In particular, approach-avoidance conflict studies reported ACC/dmPFC activation in response to trial-by-trial aversiveness (Ironside et al., 2020), and to increasing levels of conflict (Aupperle et al., 2015; McDermott et al., 2021; Schlund et al., 2016).

Beyond recognizing the importance of developing approach-avoidance conflict paradigms, it has been acknowledged that some of these paradigms should incorporate social interactions (Kirlic et al., 2017; Schultz et al., 2019). This is relevant because humans are social by nature, and social factors play a central role in mental health. For instance, some disorders (e.g., social anxiety) are defined in social terms. For other disorders such as depression, the link with social factors is less explicit, but there is no doubt they entail substantial interpersonal difficulties (Fernández-Theoduloz et al., 2019; Joiner & Timmons, 2009; Kupferberg et al., 2016). Crucially, social stressors may play an important role in driving avoidance, even when such avoidance comes at the expense of personal goals, thereby functioning as the aversive component in approach-avoidance conflict. Among these stressors, fear of negative evaluation represents a particularly salient driver in internalizing disorders such as social anxiety and depression. Defined as apprehension and distress about being judged unfavourably by others (Watson & Friend, 1969), it encompasses fears of criticism, embarrassment, and rejection, and is a robust predictor of social avoidance (Gao et al., 2025). Excessive fear of negative evaluation is a core symptom of social anxiety (Clark & Wells, 1995) and is also implicated in depression (Lucero et al., 2022).

In the present study, we aimed to develop a *social approach-avoidance conflict* (SAAC) task. In the SAAC task, participants are told that at the end of the experiment, they will have to deliver a speech to an audience of “judges,” and that their choices during the task will determine the composition of this audience. Specifically, depending on their decisions, the audience could be composed of more (higher rank) or less (lower rank) demanding judges. Each trial paired a potential reward with a judge of a particular rank. Choosing to *approach* yielded the reward but increased the presence of that judge rank in the future audience; choosing to *avoid* yielded a minimal reward but decreased the presence of that judge rank. While it would be materially optimal to approach on any trial where the reward exceeded the minimal default, individuals may instead avoid higher-rank judges because of the social evaluative demand imposed by them, even if doing so incurs associated reward costs.

Here, we used the SAAC task in combination with fMRI to examine decision-making, emotional, and neural responses driven by a social approach-avoidance conflict challenge. At the behaviour level, we hypothesized that higher rewards would promote greater approach behaviour, whereas higher judge ranks would increase avoidance and negative emotional responses. We predicted that the higher the depression and social anxiety symptoms, the less the reward would lead to approach behaviour and the more the judge rank would drive avoidance and elicit negative emotions. At the neural level, we aimed to identify brain regions responsive to the reward and judge rank during approach and avoidance decisions. Critically, we investigated how depression and social anxiety symptoms associated with modulations in these responses. We specifically expected that higher depression and social anxiety symptoms would be associated with reduced reward-related activations and enhanced aversiveness or conflict-related activations as judge rank increased.

## 2 Methods

### 2.1 Participants

The study was approved by the local research ethics committee. Written informed consent was obtained from all participants. Recruitment was conducted through the university’s social media platforms and institutional communication channels. Individuals aged 18–30 years were invited to volunteer and were directed to a study-specific website where they provided demographic information and completed the Beck Depression Inventory–II (BDI-II) (Beck et al., 1996; Sanz et al., 2003). Potential participants were assessed in a telephone interview regarding the following inclusion criteria: age between 18 and 30 years, and no use of psychiatric medication for at least 3 weeks. Exclusion criteria were a history of neurological disorders, current pregnancy, or any contraindication for MRI. During this telephone interview, participants were also assessed regarding whether they knew someone who had taken part in the current study or in previous social interaction studies conducted by our group. Most participants did not know anybody who had participated in the current or previous studies. In the very few cases in which participants reported knowing someone who had taken part, the assessment indicated that they had no knowledge of the cover story (see below). A total of 934 individuals completed the online screening, and 151 participants enrolled in the study. Several participants were excluded from the final dataset for the following reasons: inability to complete the fMRI experiment (n = 5), scanner-related technical problems (n = 7), incidental neurological findings (n = 1), unreliable or inappropriate behaviour (n = 1), missing data files (n = 1), and excessive motion artifacts (n = 1). The final sample comprised 135 participants (92 female; mean age ± SD: 23.16 ± 3.34). Due to missing questionnaire data, three participants were excluded from the analyses that involved psychological measures.

### 2.2 Psychological questionnaires

After scheduling the scanning session, the following questionnaires were sent to be completed at home: the Liebowitz Social Anxiety Scale (LSAS) (Bobes et al., 1999, p. 199; Liebowitz, 1987), the Brief Fear of Negative Evaluation (FNE) Scale (Watson & Friend, 1969; Zubeidat et al., 2007), the Self-Statements during Public Speaking Scale (SSPS) (Hofmann & DiBartolo, 2000; Rivero et al., 2010), The Rejection Sensitivity Questionnaire (RSQ) (Downey & Feldman, 1996; Frías Cárdenas & Diaz Loving, 2011), and the Self-Critical Rumination Scale (SCRS) (Martínez-Sanchis et al., 2021; Smart et al., 2016). On the scanning day, participants completed the BDI-II (Beck et al., 1996; Sanz et al., 2003) a second time (after having completed it previously during online registration).

Scores from all questionnaires were significantly intercorrelated (all *p* < 0.001). As expected, all scales were positively associated (i.e., the more depressive symptoms the more social anxiety, fear of negative evaluation, rejection sensitivity, and rumination) with the exception of the SPSS, which assesses perceived self-competence in public speaking and showed negative associations with all other measures. Scores from the six questionnaires were standardized (z-scored) and subjected to a principal component analysis (PCA) to identify reliable individual differences within the multidimensional psychological dataset. The first principal component accounted for the majority of explained variance (65.3%, see Supplementary Fig. S5) and showed loading coefficients of similar magnitude and consistent sign (BDI-II: 0.41; FNE: 0.41; LSAS: 0.42; SSPS: –0.37; RSQ: 0.41; SCRS: 0.43). This first component was, therefore, used as a continuous trait index representing each participant’s position on a depression–social anxiety (D-SA) spectrum relevant to the social approach-avoidance task. These D-SA scores were used for the behavioural, emotional, and neuroimaging analyses.

### 2.3 Social approach-avoidance conflict (SAAC) task

**Fig. 1.**
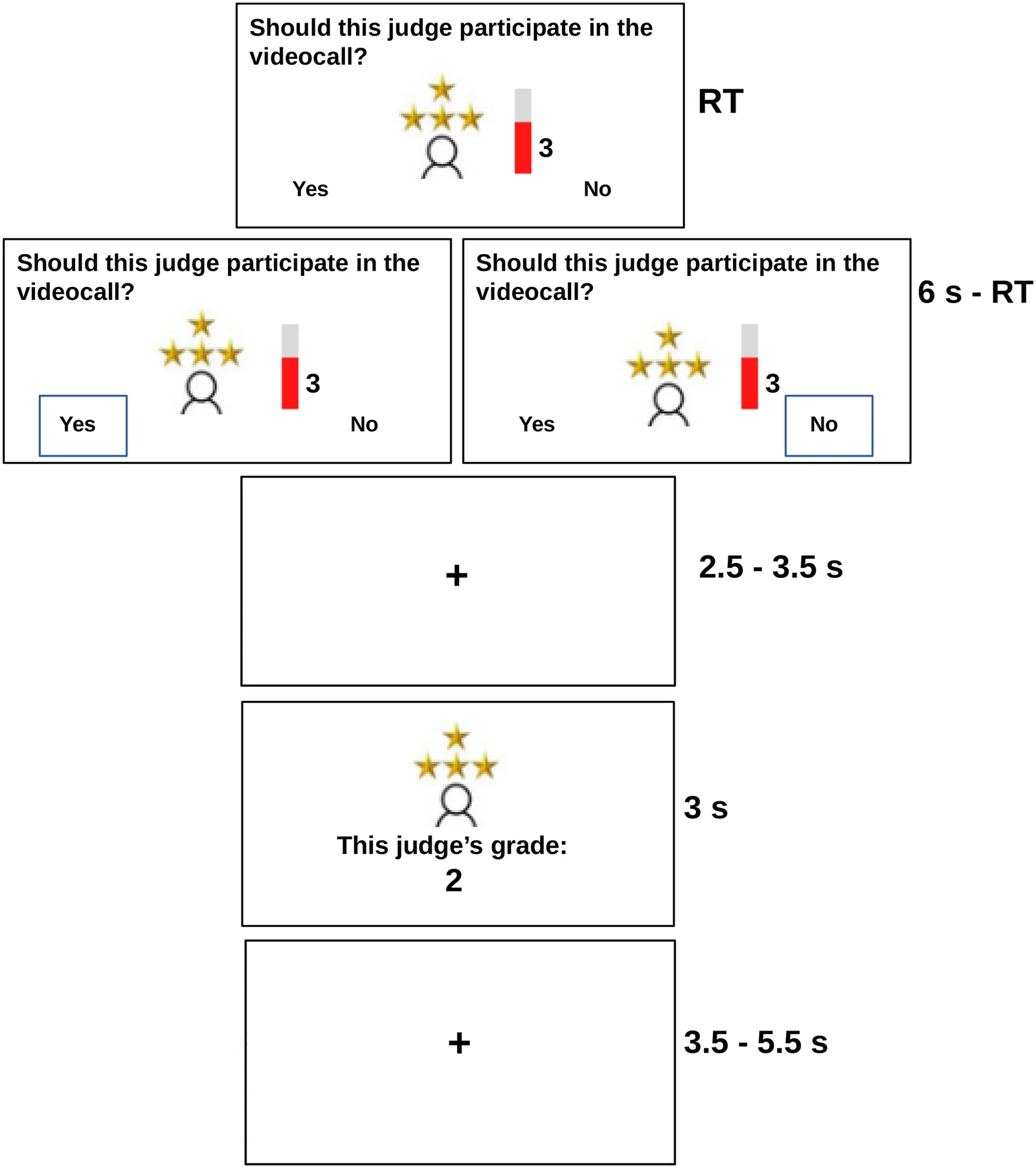
Social approach-avoidance conflict (SAAC) task. RT, reaction time; s, seconds.

During the scanning session, participants completed the SAAC task (Fig. 1). Prior to entering the scanner, they received detailed instructions. First, participants were asked to record a 2-minute audio clip in which they delivered a brief speech about a book or movie they liked. They were informed that this recording would be transmitted via a computer network to external “collaborators” who would act as judges. These judges would rate the speech on a scale from 1 to 10, with higher grades indicating better performance. In reality, no recordings were sent and no judges existed; the grades presented during scanning were predetermined.

Participants were also told that judges differed in their level of demandingness and were categorized into four groups: 1-, 2-, 3-, or 4-star judges, with higher-star judges described as more demanding. They were informed that judges would evaluate the speech based on communication and argumentation skills, and that the judges’ ranks were derived from the judges’ gradings in previous experiments. Participants were told they would receive the judges’ grades while inside the scanner. After receiving these instructions, the researcher left the room and participants were given a few minutes alone to record the audio speech. Upon returning, the researcher informed participants that the audio would now be sent to the judges for evaluation. Participants were then asked to self-rate their own speech on a 1–10 Likert scale, with 1 being bad and 10 being excellent.

Once the audio portion was completed, the remaining components of the task, taking place during and after scanning, were explained. Participants were told that after exiting the scanner, they would be required to give a second oral presentation, this time in a videocall and in the presence of several of the judges who had evaluated their audio speech. The topic for this second presentation would be revealed immediately before the videocall.

Critically, participants were instructed that during scanning they would have the opportunity to influence the composition of the final videocall audience. On each trial (Fig. 1), they viewed a silhouette representing a judge belonging to one of the four ranks (indicated by stars). Next to the silhouette, a red bar indicated the number of points (1 to 4 points) they could earn on that trial (this would be the “payment” of the trial). Participants made a binary Yes/No decision. Choosing “Yes” (approach) granted the full payment displayed but also increased the presence of that judge rank in the final videocall audience (e.g., accepting a 4-star judge increased the presence of 4-star judges in the audience). Choosing “No” (avoid) awarded 1 point by default but reduced the presence of the corresponding judge rank in the audience. Participants were informed that accumulated points would later be exchanged for a non-monetary reward, with higher totals corresponding to higher-value rewards. After each decision, a jittered fixation screen was shown, followed by presentation of the (preset) grade that the judge had allegedly assigned to their audio speech. Of note, the grade was shown in all trials, regardless of whether the participant decided to approach or avoid.

The task was implemented in PsychoPy v1.84.2 (Peirce et al., 2019) and consisted of 3 sessions of 48 trials each. In every session, each judge rank appeared 12 times, with each of the 4 possible payments presented 3 times. Judge ranks were associated with distinct grade distributions for the audio speech: 1-star judges assigned grades of 8–10; 2-star judges, 6–8; 3-star judges, 3–5; and 4-star judges, 1–3. Each individual grade appeared equally often within its judge category.

Before entering the scanner, participants completed a short practice version of the task. To strengthen the social credibility of the cover story, they were shown brief videos of supposed judges making statements such as “I’m looking forward to your presentation” or “Good luck with that.” Participants then completed a true/false questionnaire to assess comprehension of the task. In case of incorrect answers, participants were explained again the corresponding aspects of the task.

After exiting the scanner, participants completed additional emotional questionnaires (see below) and were debriefed about the cover story. No participant reported disbelief in the manipulation or expressed discomfort regarding the procedure. During debriefing, participants were asked not to discuss the cover story with future participants.

### 2.4 Emotional response questionnaires

After the scanning session, participants completed a series of ratings assessing the emotions elicited by the task. First, they indicated on discrete Likert scales (range: 0 “none” to 8 “extreme”) the intensities of happiness, relief, embarrassment, nervousness, sadness, and anger they had experienced when confronted with each judge rank during the task. They then similarly rated the intensities of happiness, relief, embarrassment, nervousness, sadness, anger, disappointment, and rejection they felt in response to 2-point binned grade feedback levels they received for their audio speech (i.e., grades 1–2, 3–4, and so on).

Participants further evaluated how much happiness, relief, embarrassment, nervousness, sadness, anger, disappointment, and rejection they experienced while performing the task, knowing that they would later deliver a speech during the videocall session. In addition, they rated how well they believed they would perform in that upcoming videocall presentation (“very bad,” “bad,” “good,” “very good”) and indicated which judge rank they preferred to be more strongly represented in the videocall audience.

### 2.5 Analysis of emotional responses

Emotional and decision-making analyses were implemented using R Statistical Software (v4.4.1; R Core Team, 2021). Emotional reports were used to fit general linear models using the glmer function (Bates et al., 2015). For analyses examining emotions elicited by judge ranks, fixed effects included judge rank, D-SA scores, sex, and age, while participant was modelled as a random effect. For analyses focused on emotions elicited by the received grades, fixed effects were grade range, D-SA scores, sex, and age with participant as a random effect.

### 2.6 Behavioural analysis

Participants’ decisions during the task were analysed using generalized linear mixed-effects models (GLMMs). The dependent variable was the binary choice on each trial (Yes = approach; No = avoidance). Judge rank, payment, D-SA scores, sex, and age were entered as fixed effects and random intercepts for participants as well as random slopes for judge rank and payment. Model significance was assessed using ANOVA-like Type II χ² Wald tests. When pairwise comparisons were required, post-hoc paired *t*-tests were conducted with Tukey’s correction for multiple comparisons.

### 2.7 fMRI image acquisition and data analyses

T2*-weighted gradient echo planar images were acquired using a 3T, GE Discovery 750 W MRI scanner with a 24-channel head coil. SPM12 (Statistical Parametric Mapping, http://www.fil.ion.ucl.ac.uk/spm) was used for analyses. Three sessions of 321 volumes were obtained with a repetition time (TR) of 2.5 seconds, echo time of 30 milliseconds, flip angle of 90°, field of view (FOV) 224 mm, and a 64 × 64 matrix. A total of 35 slices of thickness 3.6 mm and slice gap 0.4 mm were obtained for each volume.

The first four volumes were discarded to allow for scanner transient effects. For preprocessing, the first image from each session was aligned to the first scan of the first session. Then the images from each session were aligned to the first image of the session. The average realigned image was used to derive parameters for spatial normalization to the SPM12 Montreal Neurological Institute (MNI) template with the parameters applied to each image in the time series. The realigned and spatially normalized images were then smoothed with a 6-mm full-width at half-maximum (FWHM) Gaussian kernel.

For the first-level fMRI analyses, an event-related general linear model (GLM) was implemented. The onsets of approach and avoidance decisions were modelled as separate delta functions. Each regressor was further parametrically modulated by two modulators entered without orthogonalization: (i) judge rank and (ii) payment. The onset of the feedback event (i.e., the grade allegedly assigned by the judge) was modelled as an additional delta function, with a parametric modulator corresponding to the grade received. Six head-motion realignment parameters were included as nuisance regressors. All regressors of interest were convolved with the canonical haemodynamic response function (HRF) in SPM12, without inclusion of temporal or dispersion derivatives.

Second-level analyses were conducted on the resulting participant-specific beta images. To examine neural responses associated with judge rank and payment during approach and avoidance decisions, we submitted contrast images to one-sample *t*-tests. To investigate associations between individual differences in psychological traits and brain activity, contrast images were entered into second-level linear regression models with D-SA scores included as a covariate of interest. Age and sex were entered as covariates of no interest. In addition, for completeness, we performed contrasts between approach and avoidance decisions irrespective of judge rank and payment (see Supplementary Results and Supplementary Fig. S6). Unless otherwise stated, regions are reported as significant at a whole-brain *p* < 0.05 cluster level. This was achieved by a simultaneous requirement for a voxel threshold of *p* < 0.01 and a minimum cluster size of 105 continuous resampled voxels. These parameters were identified using a Monte Carlo method that simulates whole-brain fMRI activation, assumes a type I error voxel activation based on the voxel threshold, smooths the volume with a Gaussian kernel, and then counts the number of voxel clusters of a given size. After 10,000 iterations, a probability associated with each cluster extent was calculated, and we selected that which yields the desired correction for multiple comparisons (Slotnick et al., 2003).

To further assess the reliability of findings concerning regional activity as a function of psychopathology scores, post-hoc analyses were conducted on the significant clusters of interest. First, 95% confidence intervals were estimated using bootstrap resampling, with *B* = 10,000 resamplings of the association between cluster activity (beta values) and D-SA scores. Second, exact *p*-values for the association between cluster activity and D-SA scores were calculated using *B* label-shifted samples, in which the correspondence between participants’ D-SA scores and neural data was randomly permuted. Additionally, we conducted a supplementary leave-one-subject-out (LOSO) analysis to reduce potential inflation of effect sizes arising from non-independence in subsequent statistical testing (Esterman et al., 2010); see Supplementary Methods for further details on the LOSO analysis).

## 3 Results

### 3.1 Decision-making results

**Fig. 2.**
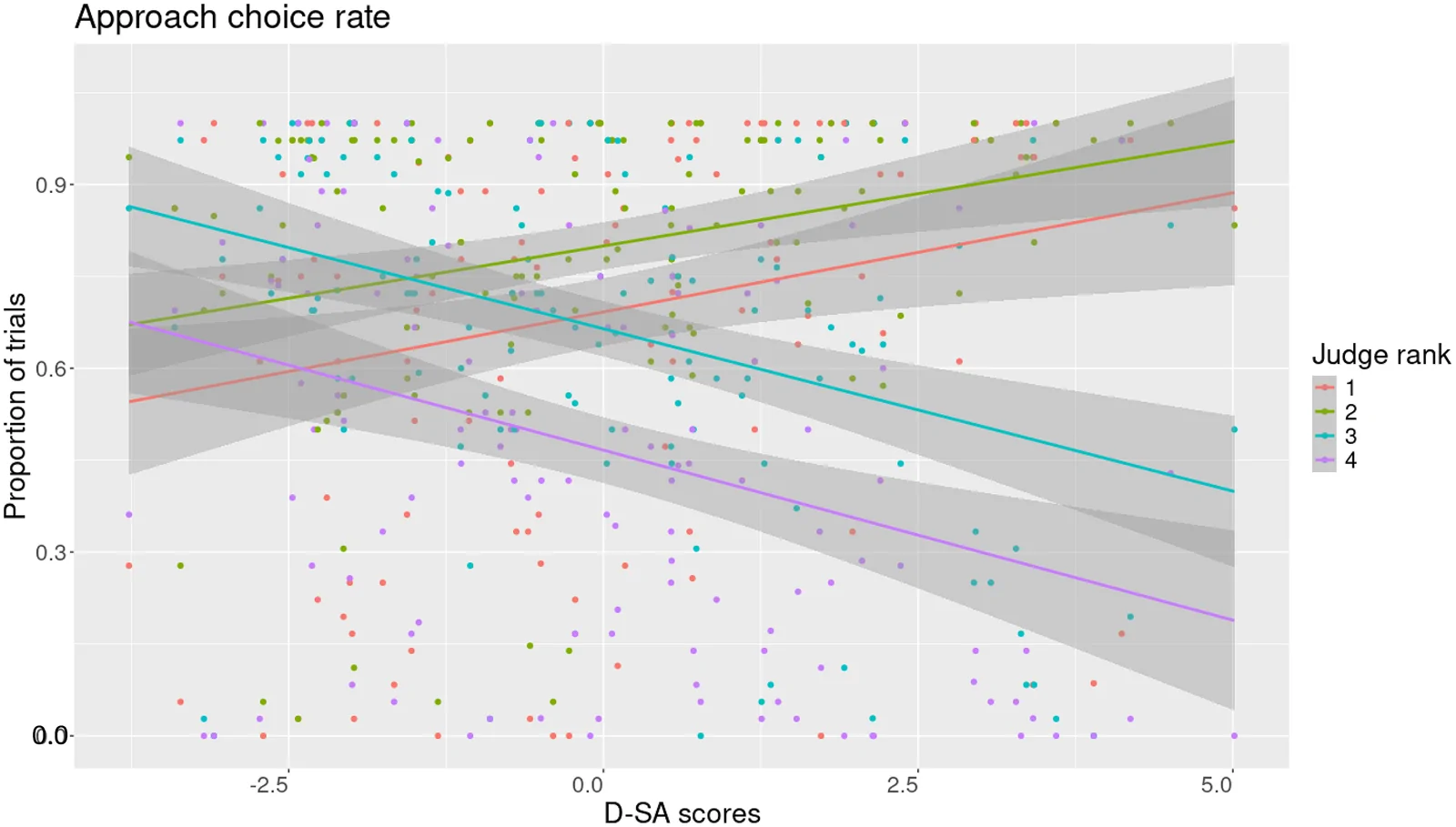
Behavioural results. Proportion of trials with approach choices as a function of participants’ depression–social anxiety (D-SA) scores, separately for each judge rank. Higher D-SA scores were associated with increased approach towards low-rank judges and increased avoidance towards high-rank judges.

The GLMM revealed a significant main effect of judge rank on participants’ choices (*χ²(3, N = *132*) = *70.8*, p < *0.001; Supplementary Table S1) (Fig. 2). Participants most frequently approached 2-star judges, closely followed by 1-star judges, although this difference did not reach significance (1-star versus 2-star, *p* = 0.066), followed by 3-star judges (2-star versus 3-star, *p* < 0.001), and finally by 4-star judges (3-star versus 4-star, *p* < 0.001; Supplementary Table S2). There was also a significant main effect of payment (*χ²(3, N = *132*)=*150*, p < *0.001), such that higher payments elicited progressively more approach decisions (all *p < *0.001; Supplementary Fig. S1). Judge rank and payment interacted significantly (*χ²(9, N = *132*) =* 28.6*, p < *0.001), with higher judge categories producing greater differentiation in approach behaviour across payment levels (Supplementary Fig. S1). Although D-SA scores did not show a significant main effect, they exhibited a significant interaction with judge rank (*χ^2^(3, N = *132*) = *24.2*, p < *0.001). Higher D-SA scores were associated with increased approach towards low rank judges (1- and 2-star; *p* < 0.001) and increased avoidance of high-rank judges (3- and 4-star; *p* < 0.001) (Fig. 2). There was not a significant interaction between D-SA scores and payment. The three-way interaction between judge rank, payment, and D-SA scores was significant (*χ²(9, N = *132*) = *16.9*, p = *0.050). Follow-up analyses indicated that, within each judge rank, payment generally did not substantially modulate the association between D-SA scores and approach/avoidance behaviour, with the exception of the highest judge rank (4-star). Specifically, for 4-star judges, higher D-SA scores were associated with greater avoidance across all payment levels except for the 1-point payment condition (Supplementary Fig. S2). In terms of total points earned during the task, there was a non-significant trend for participants with higher D-SA scores accumulating less points (*ρ = -*0.158*, p = *0.070) (see Supplementary Table S1 for full model coefficients, OR, and CIs).

### 3.2 Emotional response results

**Fig. 3.**
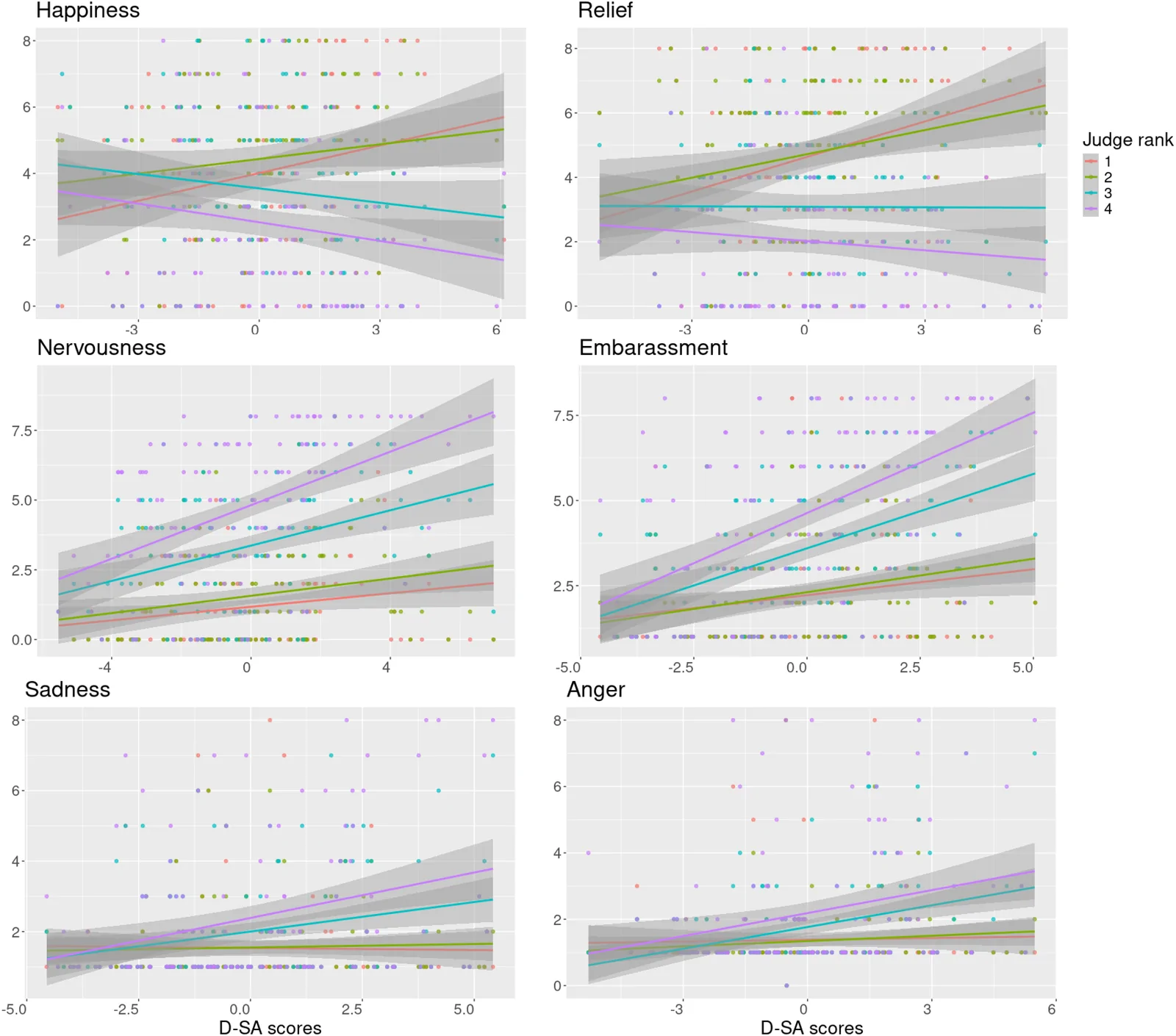
Emotional response results. Emotional ratings reported by participants as a function of judge rank and participants’ depression–social anxiety (D-SA) scores. Higher judge ranks elicited lower positive emotional responses (happiness and relief) and higher negative emotional responses (nervousness, embarrassment, sadness, and anger). Higher D-SA scores were associated with reduced positive and heightened negative emotional responses as judge rank increased.

At the end of the scanning session, participants rated their emotional responses (happiness, relief, nervousness, embarrassment, sadness, and anger) to each judge rank (Fig. 3). For all emotions, there was a significant main effect of the judge rank (*F(3,132)≥20, p < *0.001): higher judge ranks elicited higher ratings of nervousness, embarrassment, sadness, and anger, and lower ratings of happiness and relief.

A significant main effect of D-SA scores was observed for happiness, relief, and embarrassment (*F(1,132) ≥ *7.01*, p < *0.009), with higher symptom levels associated with greater embarrassment and reduced happiness and relief. For all six emotions, there was also a significant interaction between the judge rank and D-SA scores (*F(3, 132) ≥ *4.91*, p < *0.003). For happiness, higher D-SA scores predicted higher happiness ratings for 1-star judges (*t(364) = *4.24*, p < *0.001) and showed a trend towards the same pattern for 2-star judges (*t(364) = *1.88*, p = *0.061). In contrast, higher D-SA scores predicted lower happiness for 3-star and 4-star judges (*t(364) < –*2.50*, p < *0.013). For relief, higher D-SA scores predicted greater relief for 1-star and 2-star judges (*t(386) > *2.88*, p < *0.005), with no significant associations for 3-star or 4-star judges (*t(386) < –*1.57*, p > *0.117). Higher D-SA scores predicted greater nervousness for 2-star, 3-star, and 4-star judges (*t(280)>2.0, p < *0.046), but not for 1-star judges. Pairwise comparisons confirmed that slopes differed significantly across the 2-, 3-, and 4-star ranks (*t(387) > *2.92*, p < *0.020), indicating that the association of D-SA symptoms with nervousness was amplified for highest rank judges. For embarrassment, D-SA scores were positively associated with embarrassment for all judge ranks (*t(268) > *2.64*, p < *0.009), with stronger effects for 3-star and 4-star judges than for 1-star and 2-star judges (*t(387)=–*3.581*, p < *0.003). For sadness, higher D-SA scores predicted greater sadness for 3-star and 4-star judges (*t(278) > *4.03*, p < *0.001), but no significant associations were found for the 1-star or 2-star categories. For anger, higher D-SA scores similarly predicted increases for 3-star and 4-star judges (*t(301) > *3.64*, p < *0.001), with no significant associations for 1-star or 2-star judges. Taken together, across emotions, the general pattern was that higher D-SA symptoms were associated with diminished positive emotional responses and heightened negative emotional responses, particularly as judge rank increased.

Participants also rated their anticipatory emotions regarding the upcoming videocall presentation, during which they believed they would deliver a speech in front of a panel of judges. Higher D-SA scores were significantly associated with greater nervousness (*ρ = *0.463*, p < *0.001), rejection (*ρ =* 0.256*, p = *0.025), and embarrassment feelings (*ρ =* 0.606*, p < *0.001) related to the forthcoming presentation. D-SA associations were not significant (*p > *0.08) with any of the other emotional reports (happiness, relief, anger, sadness, or disappointment feelings) in connection to the anticipated task. Participants additionally rated their perceived performance in the initial audio speech (immediately after recording and again after scanning) as well as their expected performance in the videocall presentation. Higher D-SA scores were negatively associated with all three self-assessments (audio speech: *ρ = –*0.428*, p < *0.001 and *ρ = –*0.417*, p < *0.001; expected videocall performance: *ρ = –*0.549*, p < *0.001). Finally, higher D-SA scores were associated with stronger preferences for low-rank judges in the anticipated videocall audience (*ρ = –*0.479*, p < *0.001).

In addition, participants rated their emotions in response to the grades they received. As expected, higher grades elicited more positive emotions (happiness, relief), whereas lower grades elicited stronger negative emotions (nervousness, embarrassment, sadness, anger, rejection, disappointment). Across emotions, higher D-SA scores were consistently associated with heightened negative affect in response to lower grades (see Supplementary Material and Supplementary Fig. S3 for detailed results and statistics).

### 3.3 Neuroimaging results

#### 3.3.1 Activations related to the judge rank

**Fig. 4.**
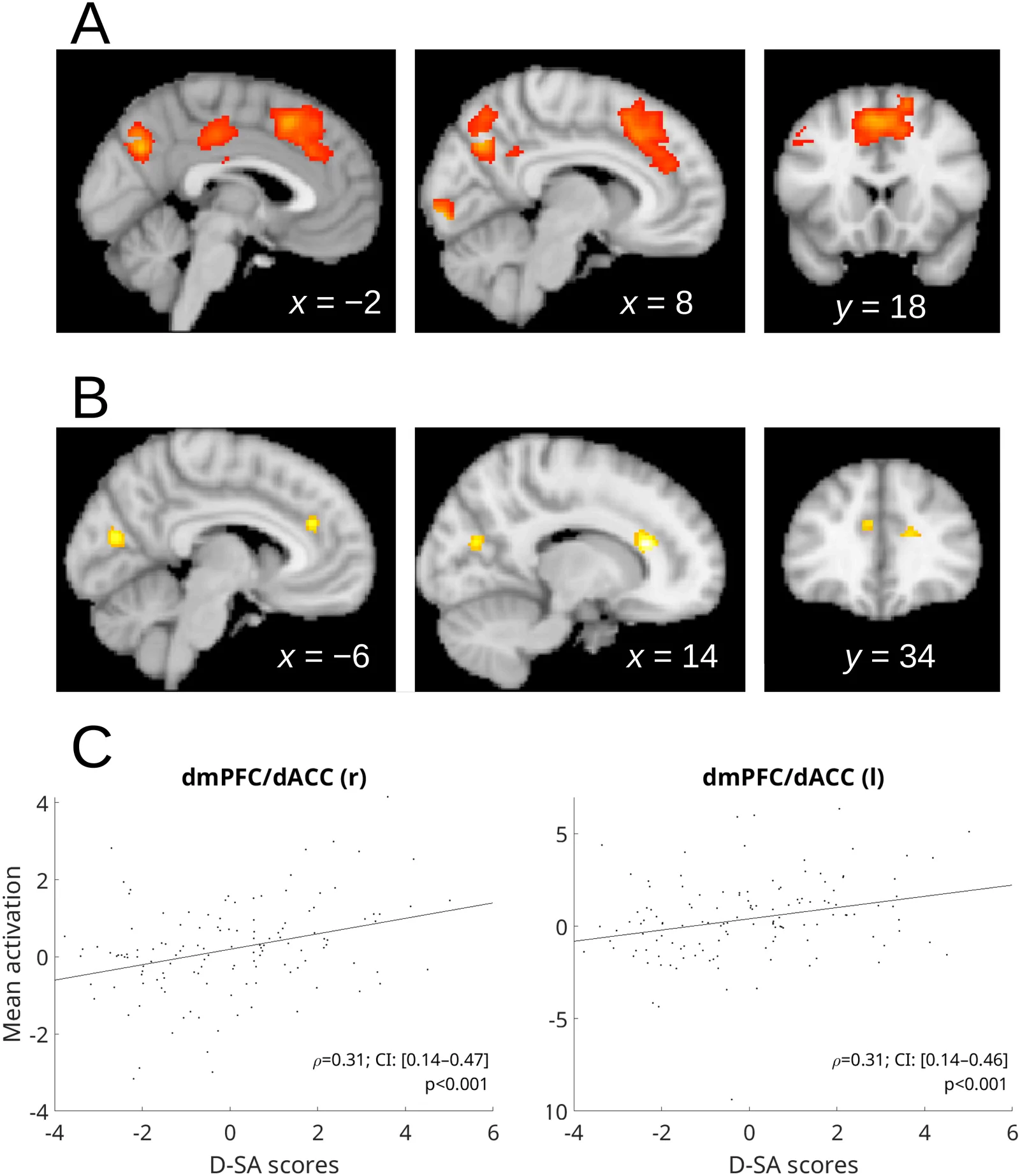
(A) Parametric activation by increasing judge rank during approach trials. (B) Brain regions with a stronger response to the increasing judge rank as the D-SA scores increased. (C) Positive correlation between D-SA scores and dmPFC/dACC activation related to increasing judge rank during approach trials. Insets indicate the rank correlation between D-SA scores and cluster activity estimates, with 95% confidence intervals and exact *p*-values.

During approach decisions, increasing judge rank elicited significant activation in the dmPFC/dACC [*(−2,18,48), t = *4.77], along with additional regions (Fig. 4A, Supplementary Table S3). During avoidance decisions, increasing judge rank again recruited the dACC [*(4,20,26), t = *3.33], as well as other areas (Supplementary Fig. S4, Supplementary Table S3). The reverse contrast (decreasing judge category) yielded comparatively weaker and more spatially restricted activations for both approach and avoidance conditions (Supplementary Table S3).

Crucially, individual differences in D-SA symptoms were associated with variation in neural responses to judge rank during approach decisions (Fig. 4B, C). Higher D-SA scores were associated with stronger dMPFC/dACC responses to increasing judge rank, with significant clusters at [*(14,28,18), t = *4.49] and [*(-6,36,26), t = *3.20] (Supplementary Table S4). For each of the two clusters, bootstrap analyses confirmed the significant association between D-SA scores and activity estimates (right: *p < *0.001, ρ CI = [0.14 – 0.47]; left: *p < *0.001, ρ CI = [0.14 – 0.46]). Furthermore, a cross-validation leave-one-subject-out (LOSO) procedure (see Section 2 and Supplementary Material) replicated the significant association between D-SA scores and right dMPFC/dACC activity (p_LOSO_ = 0.009, ρ_LOSO_ CI = [0.027 – 0.376]). The association between D-SA scores and left dMPFC/dACC activity was not replicated in the LOSO analysis (p_LOSO_ = 0.078, ρ_LOSO_ CI = [*−*0.048 – 0.296]; see Supplementary Methods). During avoidance decisions, D-SA scores were not significantly associated with modulations in dMPFC/dACC responses to judge rank (Supplementary Table S4).

#### 3.3.2 Activations related to the payment

**Fig. 5.**
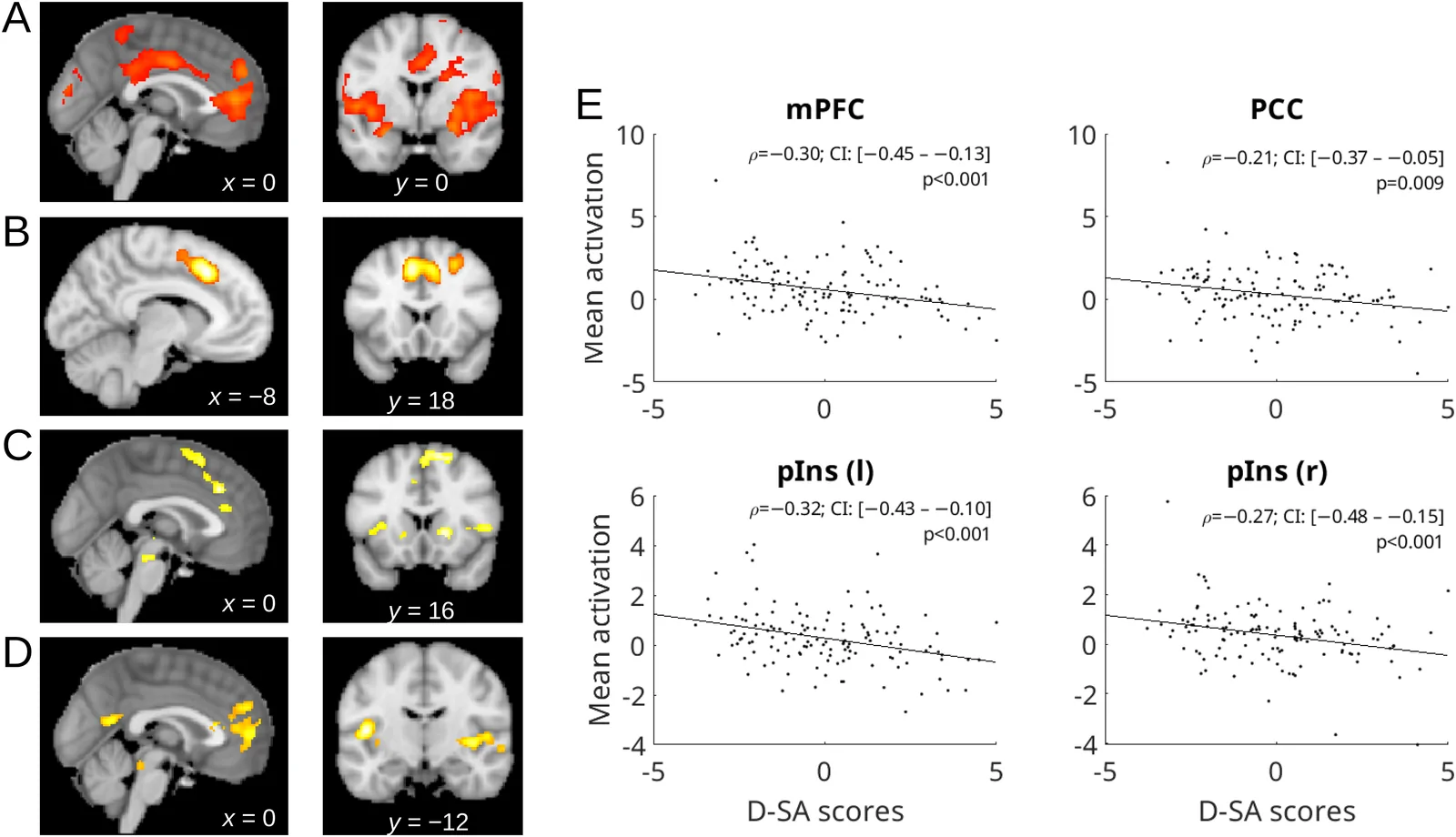
(A) Parametric activation by increasing payment during approach trials. (B) Parametric activation by decreasing payment during approach trials. (C) Parametric activation by increasing payment during avoidance trials. (D) Brain regions showing diminished response to increasing payment during approaches as the D-SA scores increased. (E) Negative correlation between D-SA scores and activation related to increasing payment during approach trials. Insets indicate the rank correlation between D-SA scores and cluster activity estimates, with 95% confidence intervals and exact *p*-values.

During approach decisions, increasing payment was associated with activation across the dorsomedial and ventromedial PFC (dmPFC/vmPFC) [*(-6,52,6), t = *4.38], posterior cingulate cortex (PCC) [*(0,−8,40), t = *4.14], and bilateral posterior insula [*(-44,4,6), t = *4.53; *(40,2,10), t = *4.35] (Fig. 5A, Supplementary Table S5). The opposite contrast (decreasing payment) revealed increased activation in the upper dmPFC [(−8,18,48), *t = *6.12] (Fig. 5B, Supplementary Table S6).

During avoidance decisions, increasing payment elicited significant activation in the dmPFC/dACC [*(6,26,32), t = *3.67] and bilateral anterior insula [*(50,16,4), t = *3.01*; (−32,16,6), t = *2.78] (Fig. 5C, Supplementary Table S5). No significant clusters emerged for decreasing payment during avoidance.

Interestingly, D-SA symptoms were significantly associated with modulations in neural responses to payment during approach decisions (Fig. 5D, Supplementary Table S6). Higher D-SA scores were associated with attenuated responses to increasing payment in the dmPFC/vmPFC [*(4,58,10), t = *3.77], PCC [*(4,−58,16), t = *3.52] and bilateral posterior insula [*(38,−12,0), t = *3.74*; (−46,−12,10), t = *4.60]. For each of the four clusters, bootstrap analyses confirmed the significant association between D-SA symptoms and activity estimates (dmPFC/vmPFC: *p* < 0.001, ρ CI = [*−*0.45 – *−*0.13]; PCC: *p *= 0.009, ρ CI = [*−*0.37 – *−*0.05]; right posterior insula: *p* < 0.001, ρ CI = [*−*0.48 – *−*0.15]; left posterior insula: *p* < 0.001, ρ CI = [*−*0.43 – *−*0.10]) (Fig. 5E). The cross-validation leave-one-subject-out (LOSO) analysis replicated the significant associations between D-SA scores and activity in the dmPFC/vmPFC (*p*_LOSO_ = 0.012, ρ_LOSO_ CI = [*−*0.36 – *−*0.03]), left posterior insula (*p*_LOSO_ = 0.003, ρ_LOSO_ CI = [*−*0.39 – *−*0.05]), and PCC (*p*_LOSO_ = 0.028, ρ_LOSO_ CI = [*−*0.32 – *−*0.01]). The LOSO analysis did not replicate the right posterior insula association with D-SA scores (*p*_LOSO_ = 0.085, ρ_LOSO_ CI = [*−*0.29 – 0.05]; see Supplementary Materials).

## 4 Discussion

We investigated decision-making, emotional responses, and neural activity in the context of a social approach-avoidance conflict, and how depression and social anxiety symptoms are associated with modulations in these processes. To this end, we designed the SAAC task, in which participants had to decide on each trial whether to approach and obtain a reward or avoid and get minimal payment. Critically, choosing to accept the reward also increased the presence of a given judge rank in a future audience who would evaluate participants in an oral presentation. Given the social evaluative demand imposed, particularly by higher-rank judges, participants might choose to avoid them, even at the cost of forfeiting rewards.

Participants’ behavioural and emotional responses indicated that, to a substantial extent, the task worked as expected. Higher payments elicited more approach behaviour, suggesting that payment functioned as an effective reward signal. In addition, higher judge ranks were associated with stronger negative and weaker positive emotional ratings, consistent with an aversive effect of judge rank. We found that behaviourally, there was no strictly monotonic increase in avoidance with increasing judge rank. While avoidance increased from 2- to 3- to 4-star judges, it was not the case that 1-star judges were the least avoided (more approached) judges. One possibility is that social desirability (not wanting to appear as someone who selects the “easiest” judges) shifted preferences towards 2-star judges. Alternatively, judge rank may also have carried some appetitive effect, such that a more exigent audience was experienced as rewarding for some participants. Nonetheless, at the upper end of the hierarchy, the aversive nature of the manipulation was evident, with 4-star judges being the most avoided. Overall, these findings suggest that, to a significant extent, the task successfully instantiated a social approach-avoidance conflict in which payment and judge rank operated as opposing motivational forces.

Importantly, depression and social anxiety symptoms related to participants’ affective and behavioural responses. Higher D-SA scores were associated with reduced positive and heightened negative emotional responses as judge rank increased. Moreover, higher D-SA scores predicted a stronger preference for low-rank judges and greater avoidance of high-rank judges. These findings are consistent with the notion that depression and social anxiety involve heightened fears of negative evaluation (Clark & Wells, 1995; Lucero et al., 2022). More broadly, the task could capture ecologically relevant situations in which individuals with internalizing symptoms forgo rewarding opportunities because they anticipate a social evaluative threat and associated distress.

### 4.1 Activations related to the judge rank

At the neural level, we observed that during approach decisions, activity in the dmPFC/dACC increased with judge rank. The dmPFC/dACC has long been related to the encoding of aversion, negative affect, and pain (Jensen et al., 2016; Journée et al., 2023; Shackman et al., 2011; Xiao & Zhang, 2018; Xu et al., 2020). In particular, this region has been implicated in social pain (Eisenberger, 2015; Rotge et al., 2015), showing activation during social exclusion (Eisenberger et al., 2003), social rejection (Fisher et al., 2010; Kross et al., 2007, 2011), and grief (Gündel et al., 2003). Moreover, the dmPFC/dACC has been reported to respond to increasing aversiveness in an approach-avoidance conflict paradigm (Ironside et al., 2020). In light of this literature, it is plausible that the dmPFC/dACC activation observed here reflects the aversive aspects of facing higher-rank judges.

A complementary interpretation is that dmPFC/dACC responses to increasing judge rank reflect the signalling of conflict and the need for cognitive control. A substantial body of work (Botvinick, 2007; Botvinick et al., 2001; Carter & Van Veen, 2007; Clairis & Lopez-Persem, 2023; Shenhav et al., 2013) links dmPFC/dACC activity to conflict monitoring and to computations related to the expected value of control. Consistent with this view, approach-avoidance conflict studies have reported dmPFC/dACC activation during high-conflict conditions (Aupperle et al., 2015; McDermott et al., 2021; Schlund et al., 2016). In our task, the higher the judge rank, the more challenging/conflictive it becomes to approach, as it implies a higher chance of future negative evaluations. It is, therefore, plausible that increasing judge rank heightens conflict and demands for control, which are reflected in dmPFC/dACC activity.

During avoidance decisions, the dmPFC/dACC also showed increased activation with higher judge rank. At first glance, one might expect that avoidance of high-rank judges would be less conflictual and thus associated with reduced dmPFC/dACC activation, because rejecting a highly threatening audience appears to be a straightforward choice. The observed pattern may suggest that avoidance of high-rank judges is closely coupled to aversive processing, whereas avoidance of low-rank judges may be driven by alternative factors, including social desirability or a genuine preference for being evaluated by a more exigent audience, as discussed above. Further work is needed to dissociate aversive from non-aversive motives during avoidance choices and to characterize dmPFC/dACC responses under each specific case.

Crucially, neural responses to increasing judge rank in the dmPFC/dACC during approach decisions were amplified in participants with higher D-SA symptoms. This pattern suggests greater aversiveness and perceived social pain, as well as heightened conflict associated with high-rank judges among individuals with elevated depressive and social anxiety symptoms. These findings align with meta-analytic evidence indicating hyperactive dmPFC/dACC responses to negative information in depression (Hamilton et al., 2012) and increased activation in this region across functional studies of depression (Graham et al., 2013). Interestingly, the dACC is the target of neurosurgical interventions for chronic, treatment-resistant depression (Christmas & Matthews, 2015), and lesion studies provide converging support for the therapeutic benefit of targeting this region (Münchenberg et al., 2022). Moreover, dmPFC/dACC hyperactivity has been repeatedly implicated in anxiety disorders. In social anxiety, increased activation has been observed during self-referential criticism (Blair et al., 2008) and in response to threat-related but task-irrelevant stimuli (Boehme et al., 2015), as well as during anticipation of fearful faces in behaviourally inhibited individuals (Clauss et al., 2014). A review concluded that dmPFC/dACC hyperactivity to threatening stimuli is a feature of social anxiety (Duval et al., 2015). Likewise, a meta-analysis of fMRI studies of induced and pathological anxiety reported increased dmPFC/dACC activity (Chavanne & Robinson, 2021), and a large transdiagnostic meta-analysis across mood and anxiety disorders found elevated dACC activation, particularly in tasks involving affective and social processing (Janiri et al., 2020).

### 4.2 Activations related to reward

During approach decisions, increasing payment evoked robust activation in dmPFC/vmPFC, PCC, and bilateral posterior insula. This pattern is consistent with a breadth of evidence linking dmPFC/vmPFC and PCC activity to the subjective value of rewards during decision making (Bartra et al., 2013; Ben-Zion & Levy, 2025; Clithero & Rangel, 2014; Levy & Glimcher, 2011). Our observations of reward value signals in the dmPFC/vmPFC are supported by convergent findings from human, non-human primate, and rodent studies, as well as lesion work (Delgado et al., 2016; Hiser & Koenigs, 2018; Moneta et al., 2024). In the case of the PCC, while the precise role of this region in reward processes during decision-making remains to be elucidated, it is hypothesized to link reward contingencies with other contextual information, possibly relying on memory processes (Foster et al., 2023). Regarding the posterior insula, this is a primary interoceptive/somatosensory area that processes appetitive input related to food, music, and touch (Davidovic et al., 2019; Mas-Herrero et al., 2021). Interestingly, the observed co-activation of the dmPFC/vmPFC and PCC is consistent with engagement of the default mode network, which has been implicated in valuation and reward-related processes (Acikalin et al., 2017).

During approach decisions, decreasing payment was associated with increased activation in the upper dmPFC. As noted above, this region has been linked to conflict monitoring and signalling of control demands (Clairis & Lopez-Persem, 2023). This appears consistent with the notion that choosing to approach when reward is low becomes more conflictual and requires greater control resources. In contrast, during avoidance decisions, increasing payment elicited activation in dmPFC/dACC and bilateral anterior insula may indicate heightened conflict and salience processing when participants chose to let go higher rewards. Taken together, these findings suggest that both approaching low rewards and avoiding high rewards are experienced as relatively difficult and conflict-laden choices.

Most critically, during approach decisions, increasing D-SA symptoms were associated with reduced neural responses to payment in dmPFC/vmPFC, PCC, and posterior insula. This pattern is consistent with a diminished subjective valuation of rewards among participants with higher depressive and social anxiety symptoms. It converges with a substantial literature implicating reward-processing dysfunction in depression (Borsini et al., 2020; Yang et al., 2022) and social anxiety (Taylor et al., 2022). In depression, altered dmPFC/vmPFC responses to reward have been reported, although the direction of effects may vary with task context (Borsini et al., 2020; Ng et al., 2019). Reduced reward-related PCC responses have also been observed in depressed participants (Satterthwaite et al., 2015; Smoski et al., 2011). In the posterior insula, attenuated reward responses have been reported both in depression (Miller et al., 2015) and in individuals with comorbid depression and anxiety (Ren et al., 2025). In social anxiety, while the existence of abnormalities in reward processing is well recognized (Taylor et al., 2022), neural reward studies are more limited than in depression and have mostly reported altered striatal responses (Cremers et al., 2015; Richey et al., 2014). Finally, both depression and anxiety were associated with reduced striatal activation during reward feedback in the context of an approach-avoidance conflict task (McDermott et al., 2022).

### 4.3 Limitations

The study had several limitations that should be noted. First, participants were recruited from a university population, which may limit the generalizability of our findings. Second, although higher D-SA symptoms were associated with attenuated neural responses to increasing payment during approach decisions, these symptoms did not predict reduced behavioural responsiveness to the payment. Future studies with larger samples and potentially refined task parameters may be better positioned to test hypothesized reductions in reward-driven approach behaviour and brain activity in individuals with elevated D-SA symptoms under social approach-avoidance conflict. Third, although judge rank generally functioned as intended, eliciting increased negative affect and avoidance at the top of the hierarchy (4-star judges), the pattern at the lower end was less straightforward. Contrary to expectations, 1-star judges were not the most preferred category. As discussed above, social desirability concerns and/or a partially appetitive challenge component of judge rank may have shifted preferences towards 2-star judges. Future work should further refine the design of social evaluative stressors to minimize these potential confounds while preserving their role as the aversive force in an approach-avoidance conflict. Finally, although most neuroimaging findings were supported by additional bootstrap/permutation and leave-one-subject-out (LOSO) validation analyses, a traditional FWE correction at the voxel level was not applied. Therefore, particularly the neuroimaging findings involving associations with D-SA symptoms should be considered preliminary, and independent replication in future studies will be important.

## 5 Conclusion

The present study introduces a novel social approach-avoidance conflict paradigm grounded in a social evaluative demand manipulation. Our findings suggest that, for individuals with higher depressive and social anxiety symptoms, being pulled between approaching a reward and avoiding a social stressor (specifically, being evaluated by highly demanding others) is particularly challenging and characterized by heightened emotional sensitivity to social threat and increased avoidance. At the neural level, the results highlight a role for the dmPFC/dACC in signalling greater perceived aversiveness and conflict in response to the social stressor, together with attenuated reward-related activity in regions implicated in valuation. Taken together, these behavioural, emotional, and neural findings help clarify how internalizing symptoms shape the experience of social approach-avoidance conflict, favouring avoidance over engagement with potentially rewarding opportunities.

## Supplementary Material

Supplementary Material

## Data Availability

The data underlying this article cannot be shared publicly because it would require participants to sign an additional consent form, which is not possible to implement at the moment. The code used for the analysis is available at https://osf.io/uq837/
