# Supplementary material for: Social approach-avoidance conflict: Behavioural, emotional, and neural correlates along a depression–social anxiety spectrum
Source: Imaging Neurosci (Camb). 2026 Sep 10;4:IMAG.a.1363. doi: 10.1162/IMAG.a.1363 (PMC13563531; doi:10.1162/IMAG.a.1363)
Supplement: Supplementary file 1 [file IMAG.a.1363_supp.pdf]

Supplementary Methods

To address the issue of statistical non-independence, in which data selected on the basis of an initial statistical test are then subject to another increasing the likelihood of effect size inflation and false-positive rates, we conducted leave-one-subject-out (LOSO) cross-validation on correlational data. In each iteration (N=131), a single n-th subject was excluded of the second-level linear regression model group analysis run with D-SA scores as covariate of interest and with identical thresholding as reported in the main text. With fewer degrees of freedom, the n-th 'LOSO GLM' reflects a more conservative test. At each iteration, each resulting set of ROIs served as localizers that were applied to the n-th subject first-level beta weights, introducing independence. For example, suppose that in the n-th LOSO GLM a significant association between D-SA and dmPFC/vmPFC cluster activity was found in response to payment during approach decisions across N=130 participants. In this case we addressed individual n-th participant data at first-level analyses, concerning their responses to payment during approach decisions within the identified localizer at dmPFC/vmPFC. The procedure was then iterated across subjects. LOSO cluster activity was extracted from all six significant clusters determined in the original GLM (see main text Results). We further ensured that independent LOSO localizers (a) referred to the same neighborhood as the original ROIs, and (b) activity was not dominated by contributions outside of the same region. For this, we excluded LOSO localizers that (i) did not include the corresponding ROI voxel referenced in the main text, and (ii) were excessively large. Exceedance was determined by meeting two conditions, first, a size larger than three standard deviations above the average size of all LOSO localizers from the same ROI. Second, a size twice or more larger than any of the included localizers. All criteria resulted in exclusions of 5 participants in the case of the left dmPFC/dACC ROI and 6 participants in the case of the PCC ROI. For the right dmPFC/dACC, and right and left insulae ROIs, the criteria led to exclusions of 1 participant in each case. Subsequent correlation analyses were then performed per ROI using the resulting data across all subjects and their D-SA measures, computing exact p-values and CIs based on bootstrap and label-shifting methods as explained in the main text.

Supplementary Results

Emotions in response to the grade feedback

At the end of the session, participants rated their emotions (happiness, relief, nervousness, embarrassment, sadness, anger, rejection and disappointment) in response to the grade feedback they received (see Supplementary Figure 3). For simplicity, analyses were performed on grade groupings (1–2, 3–4, 5–6, 7–8, and 9–10). For every emotion, there was a significant main effect of grade ($F(4, 516) \geq 71$, $p<0.001$), such that higher grades were associated with higher ratings of happiness and relief, whereas lower grades elicited higher ratings of nervousness, embarrassment, sadness, anger, rejection, and disappointment. A significant main effect of the D-SA symptoms was found for all negative emotions ($F(1, 279) \geq 10.3$, $p<0.001$), with higher levels of symptoms being associated with more nervousness, embarrassment, sadness, anger, rejection and disappointment.

Across all emotions, a significant interaction between grade and D–SA scores emerged ($F(4, 516) \geq 3.55$, $p < 0.008$). For happiness, the interaction was driven by a significantly positive association between D-SA scores and happiness for 9–10 grades ($t(341) = 2.01$; $p=0.046$). Slopes for all other grade ranges were not significantly different from zero ($p > 0.22$).

For relief, the interaction reflected significantly positive associations for the 7–8 and 9–10 grades ($t(373) > 4.39$; $p<0.001$). Slopes for lower grade ranges were not significantly different from zero ($p>0.131$). Pairwise comparisons indicated no significant difference between the 7–8 and 9–10 slopes ($t(516)= -0.706$; $p=0.96$).

For nervousness, D–SA scores were positively associated with nervousness for grades 1–2, 3–4, and 5–6 ($t(267)>3.79$; $p<0.001$). Slopes for 7–8 and 9–10 grades were not significant ($p>0.115$). Pairwise comparisons revealed that the slope for 1–2 grades was significantly greater than that for 5–6 grades ($t(516)= 3.807$; $p=0.002$),whereas differences between 1–2 and 3–4, and between 3–4 and 5–6 grades, were not significant ($p>0.077$).

For embarrassment, positive associations with D–SA scores were observed for all grade ranges except 9–10 ($t(338) > 2.93$; $p<0.004$). The slope for 9–10 grades was not significantly different from zero ($p=0.38$). Pairwise comparisons showed significant slope differences between 1–2 or 3–4 and 7–8 grades ($t(516)> 4.61$; $p<0.001$), and also between 3–4 and 5–6 grades ($t(516) = 2.78$; $p=0.044$). All other slope differences were nonsignificant ($p>0.067$).

For sadness, D–SA scores were positively associated with sadness for grades 1–2, 3–4, and 5–6 ($t(302) > 3.90$; $p<0.001$). Slopes for 7–8 and 9–10 grades were not significant ($p>0.28$). Pairwise comparisons indicated no significant differences among the positive slopes ($p>0.092$).

For anger, positive associations with D–SA scores were observed for grades 1–2, 3–4, and 5–6 ($t(279) > 2.55$; $p<0.012$). Slopes for 7–8 and 9–10 grades were not significant ($p>0.38$). Pairwise comparisons indicated no significant differences between the positive slopes ($p>0.90$).

For rejection, the interaction was driven by significantly positive slopes for the 1–2, 3–4, and 5–6 grades ($t(345) > 4.32$; $p<0.001$). Slopes for 7–8 and 9–10 grades were not significant ($p>0.11$). Pairwise comparisons revealed no significant differences among the positive slopes ($p>0.79$).

For disappointment, D–SA scores were positively associated with disappointment for grades 1–2, 3–4, and 5–6 ($t(397) > 5.55$; $p<0.001$). Slopes for 7–8 and 9–10 grades were not significantly different from zero ($p>0.10$). Pairwise comparisons showed no significant differences among the positive slopes ($p>0.60$).

Supplementary Figures

Supplementary Figure 1 Approach rate by judge rank and payment

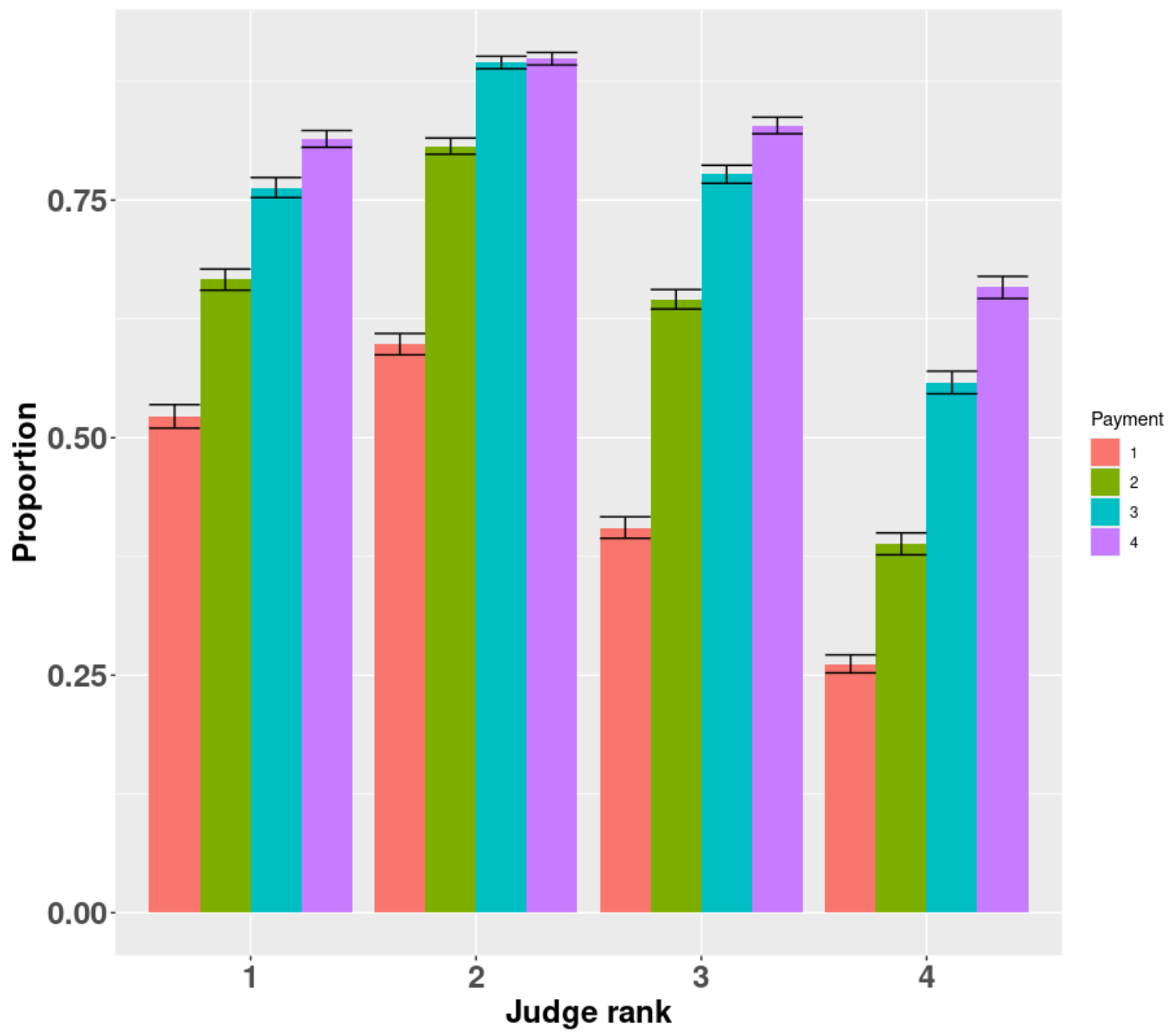

Supplementary Figure 2. Approach rate by judge rank, D-SA scores and payment. Every panel represents a different judge rank, ranging from 1 to 4-stars. Across different ranks, D-SA symptoms increase the likelihood of approach[avoidance] behavior to lower[higher] rank judges irrespective of payment offer, except for 1-point offers at the highest judge rank. In this case, participants generally avoid the offer regardless of D-SA score.

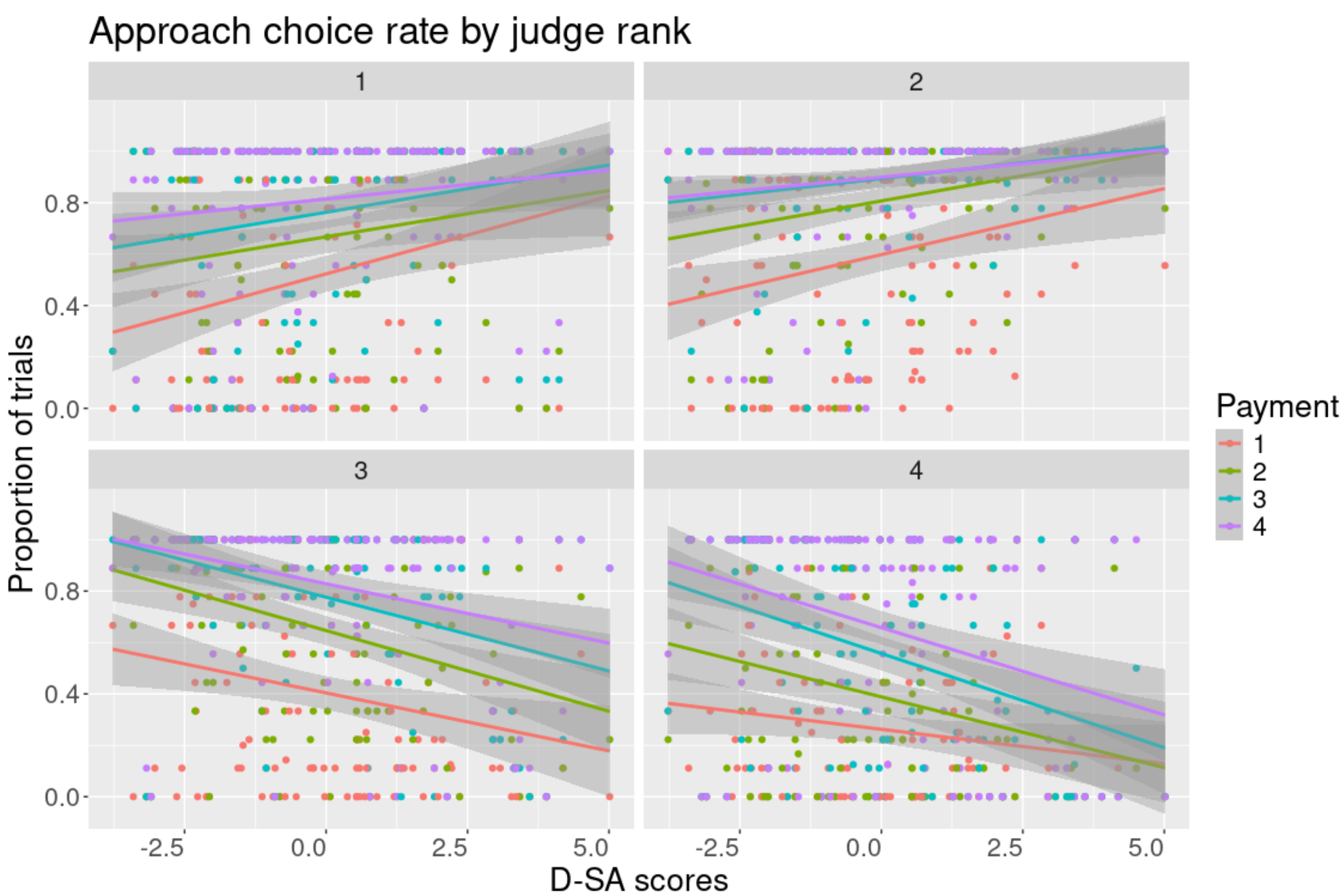

Supplementary Figure 3. Emotions related to the grade feedback

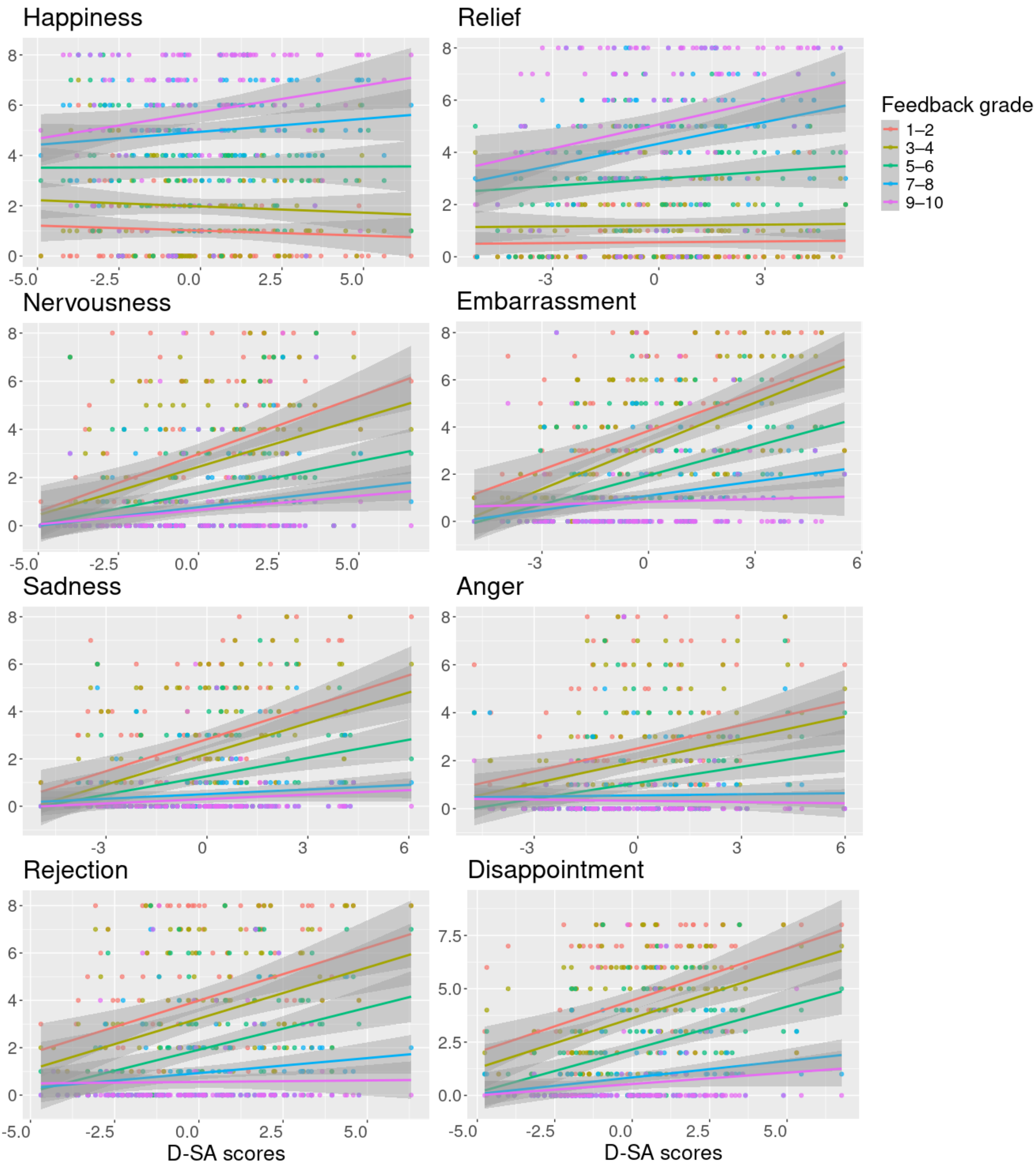

Supplementary Figure 4. Parametric activation by increasing judge rank during avoidance trials.

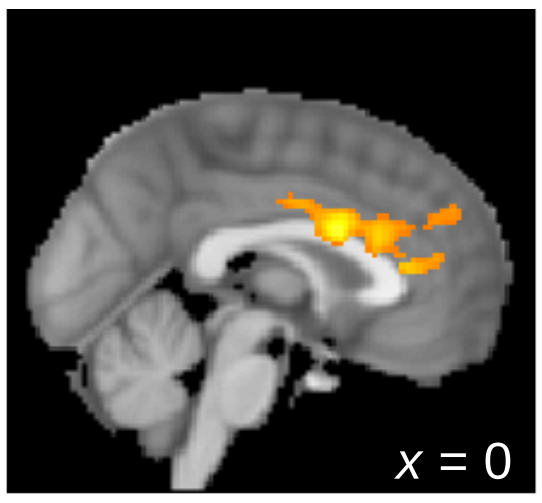

Supplementary Figure 5. Scree plot showing the explained variance of the PCA components based on questionnaire data.

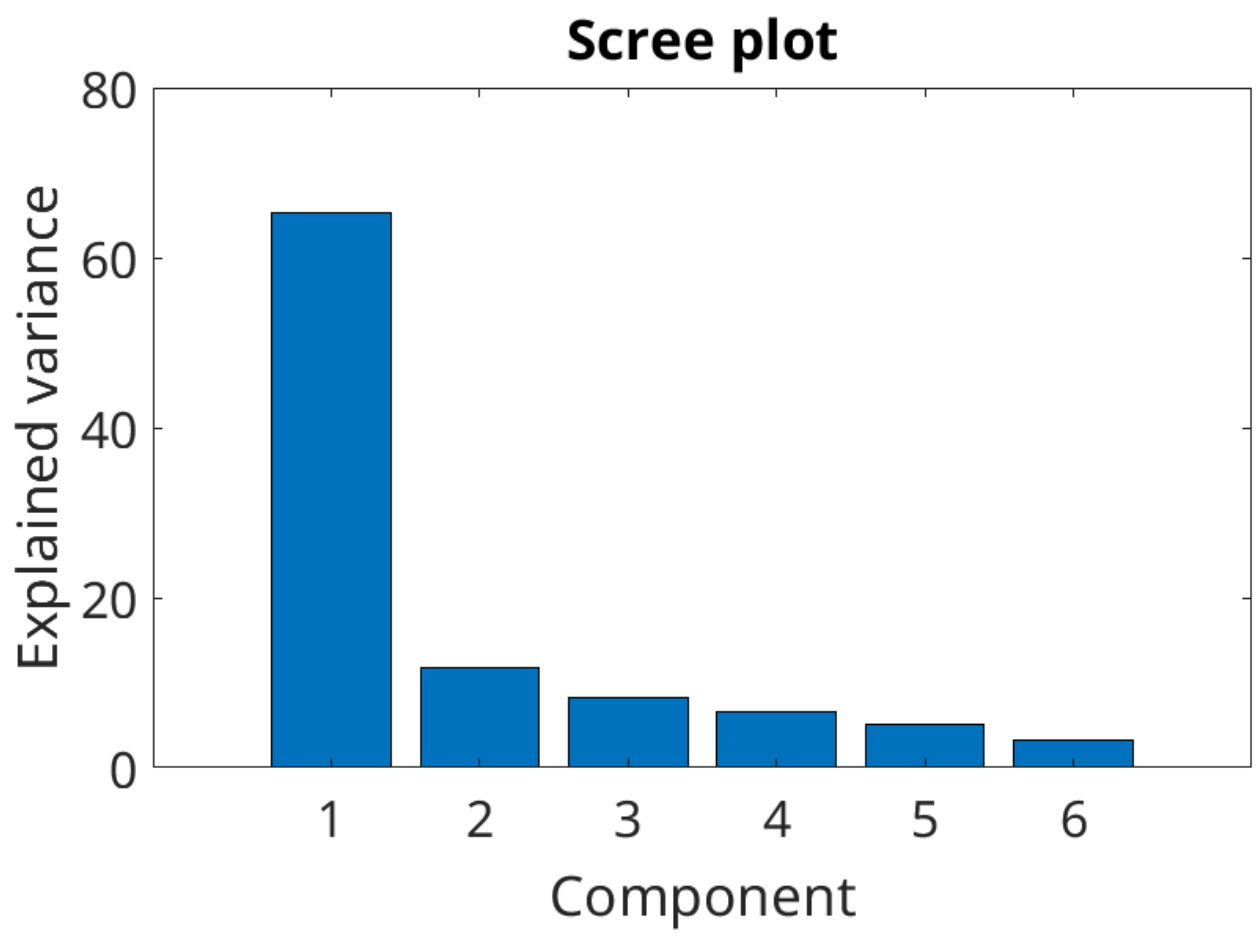

Supplementary Figure 6. Contrasts of approach versus avoidance decisions irrespective of judge rank and reward. A) For approach > avoidance contrasts, widespread activations were observed across vmPFC, PCC, dlPFC, posterior insula, thalamus, occipital cortex. B) For avoidance > approach contrasts, activations were observed in the superior frontal gyrus, frontal operculum, and angular gyrus.

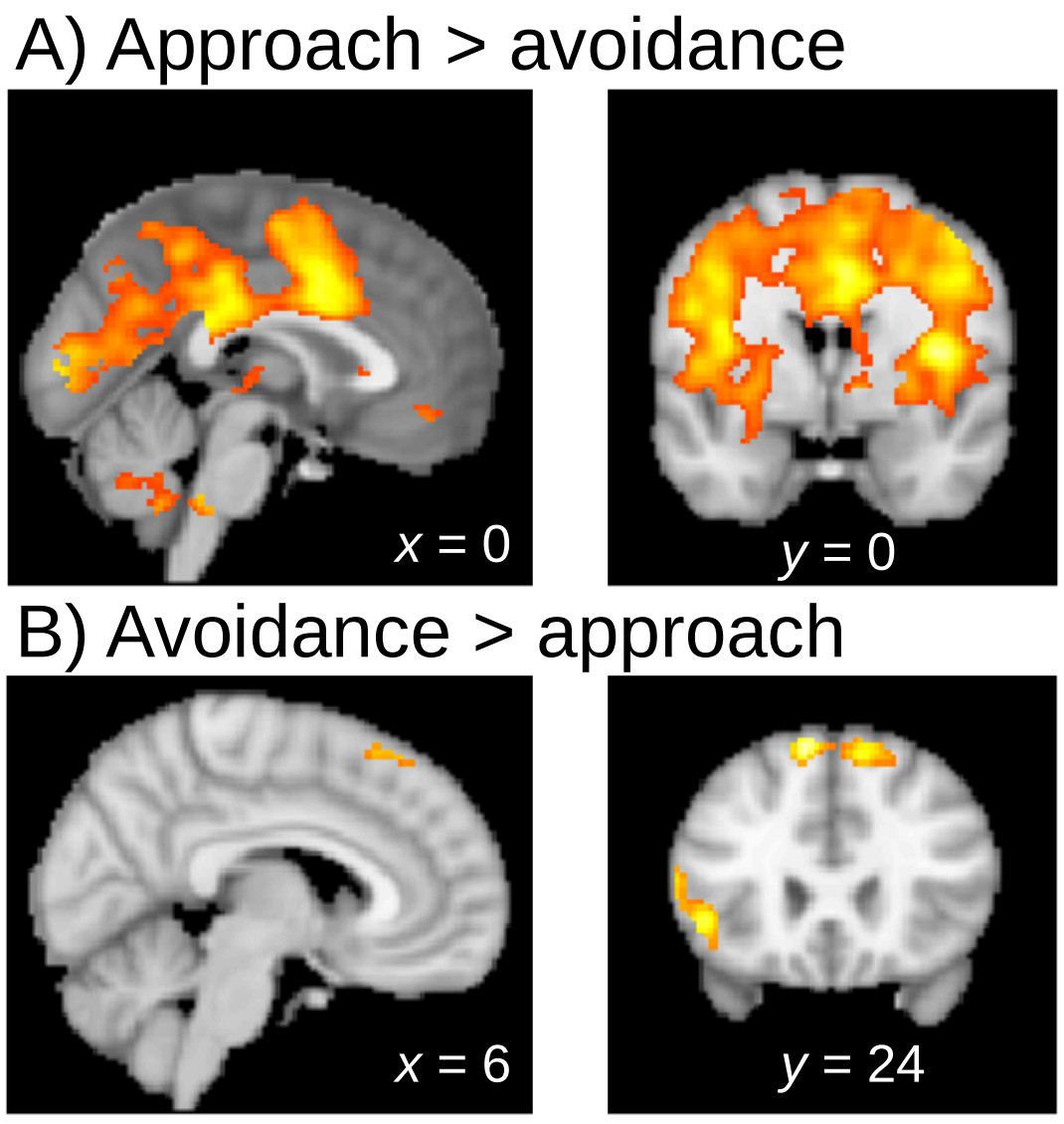

Supplementary Tables

Supplementary Table 1. Behavioral GLM coefficients with odds ratio and confidence intervals from complete model re-fit with standardized data.

Bold indicate significant odds ratios.

Parameter	***Fixed effects b***	***Standard odds ratio***	***95% CI***
**Intercept**	**1.54**	**4.65**	**[2.97, 7.28]**
D-SA	−0.07	0.87	[0.67, 1.14]
**Judge [^1]**	**−1.96**	**0.14**	**[0.07, 0.29]**
**Judge [^2]**	**−1.16**	**0.31**	**[0.21, 0.46]**
**Judge [^3]**	**0.36**	**1.43**	**[1.13, 1.83]**
**Pay [^1]**	**2.78**	**16.03**	**[10.26, 25.05]**
**Pay [^2]**	**−0.26**	**0.77**	**[0.65, 0.91]**
Pay [^3]	−0.02	0.99	[0.86, 1.13]
Sex [F]	0.12	1.13	[0.66, 1.91]
Age	−0.15	0.86	[0.68, 1.09]
**D-SA × Judge [^1]**	**−0.90**	**0.17**	**[0.08, 0.36]**
D-SA × Judge [^2]	**−0.08**	0.98	[0.67, 1.44]
**D-SA × Judge [^3]**	**0.25**	**1.64**	**[1.29, 2.08]**
**D-SA × Pay [^1]**	**−0.28**	**0.57**	**[0.37, 0.89]**
D-SA × Pay [^2]	0.06	1.12	[0.95, 1.31]
D-SA × Pay [^3]	−0.02	0.97	[0.85, 1.11]
Judge [^1] × Pay [^1]	0.07	1.07	[0.84, 1.37]
Judge [^2] × Pay [^1]	<0.01	1.00	[0.79, 1.27]
Judge [^3] × Pay [^1]	−0.21	0.81	[0.63, 1.03]
Judge [^1] × Pay [^2]	0.21	1.23	[0.99, 1.54]
**Judge [^2] × Pay [^2]**	**0.41**	**1.50**	**[1.21, 1.87]**
Judge [^3] × Pay [^2]	−0.15	0.86	[0.69, 1.08]
Judge [^1] × Pay [^3]	−0.03	0.97	[0.78, 1.19]
**Judge [^2] × Pay [^3]**	**−0.23**	**0.80**	**[0.65, 0.98]**
Judge [^3] × Pay [^3]	−0.12	0.89	[0.72, 1.10]
(D-SA × Judge [^1]) × Pay [^1]	−0.09	0.84	[0.65, 1.10]
(D-SA × Judge [^2]) × Pay [^1]	−0.11	0.80	[0.62, 1.02]
**(D-SA × Judge [^3]) × Pay [^1]**	**−0.14**	**0.75**	**[0.59, 0.97]**
(D-SA × Judge [^1]) × Pay [^2]	0.08	1.16	[0.92, 1.46]
(D-SA × Judge [^2]) × Pay [^2]	0.02	1.04	[0.83, 1.30]
(D-SA × Judge [^3]) × Pay [^2]	>−0.01	0.99	[0.79, 1.24]
(D-SA × Judge [^1]) × Pay [^3]	0.09	1.19	[0.96, 1.49]
(D-SA × Judge [^2]) × Pay [^3]	−0.06	0.89	[0.72, 1.11]
(D-SA × Judge [^3]) × Pay [^3]	0.06	1.12	[0.90, 1.39]

Supplementary Table 2. Post-hoc comparisons of approach behavior by significant manipulations of judge rank, payment., and judge rank by D-SA interaction.

**Main effect: Judge rank**	*Estimate*	*P-value*	*S. odds ratio*	*95% CI*
Rank1 − Rank2	−0.603	0.066	0.547	[0.29 – 1.03]
Rank1 − Rank3	0.760	0.303	2.137	[0.70 - 6.57]
Rank1 − Rank4	2.474	<0.001	11.868	[3.53 – 39.88]
Rank2 − Rank3	1.363	<0.001	3.907	[1.79 – 8.51]
Rank2 − Rank4	3.077	<0.001	21.695	[7.64 – 61.60]
Rank3 − Rank4	1.714	<0.001	5.552	[3.16 – 9.76]

**Main effect: Pay**				
Pay1 − Pay2	-1.492	<0.001	0.225	[0.15 – 0.34]
Pay1 − Pay3	-2.753	<0.001	0.064	[0.03 – 0.12]
Pay1 − Pay4	-3.716	<0.001	0.024	[0.01 – 0.05]
Pay2 − Pay3	-1.261	<0.001	0.283	[0.20 – 0.41]
Pay2 − Pay4	-2.224	<0.001	0.108	[0.06 – 0.19]
Pay3 − Pay4	-0.963	<0.001	0.382	[0.27 – 0.53]

**Judge x D-SA interaction**				
Rank1 − Rank2	0.170	0.208	1.185	[0.86 – 1.64]
Rank1 − Rank3	0.909	<0.001	2.483	[1.38 – 4.45]
Rank1 − Rank4	1.095	<.001	2.989	[1.58 – 5.64]
Rank2 − Rank3	0.739	<.001	2.095	[1.40 – 3.13]
Rank2 − Rank4	0.925	<.001	2.522	[1.46 – 4.35]
Rank3 − Rank4	0.186	0.208	1.204	[0.89 – 1.63]

Supplementary Table 3. Brain activations related to the judge rank in approach and avoidance decisions.

L/R, Left/Right; Coordinates (x,y,z) reported in MNI space. -a Indicates that the peak belongs to the same cluster as the peak above.

Region Name	Cluster size	X	Y	Z	T
**Approach: Increasing judge rank**					
dmPFC/dACC	2504	−2	18	48	4.77
PCC	516	0	−24	42	3.83
R Occipital fusiform gyrus	1099	20	−84	−14	6.97
L Precentral gyrus	2200	−38	−20	52	5.74
R Precuneus	1664	12	−64	36	5.17
R Angular gyrus	532	42	−64	48	4.29
L Angular gyrus	929	−34	−58	50	3.76
L Occipital pole	689	−20	−98	8	3.88
**Approach: Decreasing judge rank**					
R Posterior ínsula	264	36	−18	12	3.81
L Lingual gyrus	277	−12	−70	−8	5.14
R Precentral gyrus	208	38	−18	48	3.60
R Inferior occipital gyrus	173	48	−66	−2	3.16
**Avoidance: Increasing judge rank**					
dACC	3798	4	20	26	3.33
L Superior frontal gyrus	-a	−20	52	28	4.35
R Superior frontal gyrus	-a	20	52	28	3.42
R Anterior insula	1092	38	6	4	4.19
R Precentral gyrus	464	40	−20	62	3.96
L Cerebellum	428	−22	−48	−26	3.43
R Lingual gyrus	330	20	−90	−8	5.44

L Middle temporal gyrus	266	−54	−38	−6	3.61
R Middle temporal gyrus	200	48	−32	0	2.97
R Inferior temporal gyrus	121	46	−56	−4	3.25
L Occipital fusiform gyrus	173	−22	−86	−10	3.39
**Avoidance: Decreasing judge rank**					
L Precentral gyrus	790	−38	−24	52	4.59
L Supramarginal gyrus	143	−48	−38	50	3.09
L Supplementary motor cortex	157	−6	−6	54	3.39
R Superior parietal lobule	409	28	−46	42	3.93

Supplementary Table 4. Brain activations related to the judge rank as a function of D-SA scores in approach and avoidance choices.

L/R, Left/Right; Coordinates (x,y,z) reported in MNI space. -a Indicates that the peak belongs to the same cluster as the peak above.

Region Name	Cluster size	X	Y	Z	T
**Approach: Increasing judge rank**<br>**Increasing D-SA scores**					
R dACC	214	14	28	18	4.49
L dmPFC/dACC	137	−6	36	26	3.20
L Superior occipital gyrus	887	−26	−76	22	4.50
R Precuneus	135	4	−68	30	2.81
**Approach: Increasing judge rank**<br>**Decreasing D-SA scores**					
L Cerebellum	118	−8	−52	−34	3.55
L Superior temporal gyrus	147	−54	−2	−8	2.94
**Avoidance: Increasing judge rank**<br>**Increasing D-SA scores**					
R Cerebellum	266	28	−58	−50	4.87
L Cerebellum	224	−14	−72	−42	3.70
R Postcentral gyrus	4556	34	−32	66	4.76
L Precentral gyrus	-a	−18	−22	68	4.60
R Superior parietal lobule	-a	24	−72	50	4.04
R Mid ínsula	154	34	0	4	3.67
L Inferior temporal gyrus	219	−48	−60	−4	4.12
R Inferior temporal gyrus	196	54	−58	−12	3.52
L Pallidum	134	−20	−10	−2	3.44
R Hippocampus	113	28	−24	−6	3.13
**Avoidance: Increasing judge rank**<br>**Decreasing D-SA scores**					
R PCC	499	6	0	30	3.33

R Middle frontal gyrus	111	38	12	30	3.05

Supplementary Table 5. Brain activations related to the payment in approach and avoidance choices.

Region Name	Cluster size	X	Y	Z	T
**Approach: Increasing payment**					
dmPFC/vmPFC	3281	−6	52	6	4.38
Superior frontal gyrus (medial segment)	-a	−6	46	28	5.37
PCC	5477	0	−8	40	4.14
R Precentral gyrus	-a	28	−20	70	6.66
L Posterior ínsula	6049	−44	4	6	4.53
R Posterior ínsula	5631	40	2	10	4.35
R Inferior frontal gyrus	-a	52	34	−2	4.34
L Lingual gyrus	3009	−10	−84	−14	5.29
R Precuneus	112	20	−54	10	3.20
**Approach: Decreasing payment**					
dmPFC	1238	-8	18	48	6.12
R Lingual gyrus	845	14	−82	−12	6.45
L Precentral gyrus	1158	−34	−20	52	5.09
L Occipital fusiform gyrus	140	−30	−78	−12	5.66
R Middle frontal gyrus	343	26	18	50	4.08
L Inferior occipital gyrus	406	−16	−98	2	5.19
**Avoidance: Increasing payment**					
dmPFC/dACC	1185	6	26	32	3.67
R Superior frontal gyrus	-a	12	18	60	3.76
R Anterior insula	256	50	16	4	3.01
L Anterior insula	130	−32	16	6	2.78
Brainstem	247	−6	−28	−18	3.10
R Supramarginal gyrus	392	38	−40	38	3.41
**Avoidance: Decreasing payment**					

R Cerebellum	710	28	−72	−40	3.78
L Cerebellum	475	−26	−68	−24	3.92

Supplementary Table 6. Brain activations related to payment as a function of D-SA scores in approach and avoidance choices.

L/R, Left/Right; Coordinates (x,y,z) reported in MNI space. -a Indicates that the peak belongs to the same cluster as the peak above.

Region Name	Cluster size	X	Y	Z	T
**Approach: Increasing payment**<br>**Increasing D-SA scores**	No significant differences were observed				
**Approach: Increasing payment**<br>**Decreasing D-SA scores**					
dmPFC/vmPFC	674	4	58	10	3.77
PCC	163	4	−58	16	3.52
R Posterior ínsula	779	38	−12	0	3.74
L Posterior ínsula	873	−46	−12	10	4.60
L Parahippocampal gyrus	500	−14	−34	−10	3.31
L Lingual gyrus	-a	−16	−52	−2	2.94
R Lingual gyrus	280	12	−50	−4	3.28
L Superior frontal gyrus (medial segment)	213	−4	52	28	3.38
L Middle frontal gyrus	167	−30	34	30	3.17
R Angular gyrus	225	48	−58	22	2.70
**Avoidance: Increasing payment**<br>**Increasing D-SA scores**	No significant differences were observed				
**Avoidance: Increasing payment**<br>**Decreasing D-SA scores**					
PCC	3818	−4	−10	32	3.68
L Postcentral gyrus	124	−32	−28	66	3.72
